# Fluctuation Imaging of LRRK2 Reveals that the G2019S Mutation Alters Spatial and Membrane Dynamics

**DOI:** 10.3390/molecules25112561

**Published:** 2020-05-31

**Authors:** Bethany J. Sanstrum, Brandee M. S. S. Goo, Diana Z. Y. Holden, Donovan D. Delgado, Thien P. N. Nguyen, Kiana D. Lee, Nicholas G. James

**Affiliations:** Department of Cell and Molecular Biology, University of Hawaii at Manoa, 651 Ilalo Street, Honolulu, HI 96813, USA; BSANSTRUM@augusta.edu (B.J.S.); BGOO@augusta.edu (B.M.S.S.G.); dianazyh@berkeley.edu (D.Z.Y.H.); dddelgad@hawaii.edu (D.D.D.); thienn@hawaii.edu (T.P.N.N.); kiana.lee2@wsu.edu (K.D.L.)

**Keywords:** Parkinson’s disease, LRRK2, endocytosis, FLIM, FRET-Phasor, fluorescence fluctuation spectroscopy

## Abstract

Mutations within the *Leucine-Rich Repeat Kinase 2 (LRRK2)* gene are the most common genetic cause of autosomal and sporadic Parkinson’s disease (PD). LRRK2 is a large multidomain kinase that has reported interactions with several membrane proteins, including Rab and Endophilin, and has recently been proposed to function as a regulator of vesicular trafficking. It is unclear whether or how the spatiotemporal organization of the protein is altered due to LRRK2 activity. Therefore, we utilized fluctuation-based microscopy along with FLIM/FRET to examine the cellular properties and membrane recruitment of WT LRRK2-GFP (WT) and the PD mutant G2019S LRRK2-GFP (G2019S). We show that both variants can be separated into two distinct populations within the cytosol; a freely diffusing population associated with monomer/dimer species and a slower, likely vesicle-bound population. G2019S shows a significantly higher propensity to self-associate in both the cytosol and membrane regions when compared to WT. G2019S expression also resulted in increased hetero-interactions with Endophilin A1 (EndoA1), reduced cellular vesicles, and altered clathrin puncta dynamics associated with the plasma membrane. This finding was associated with a reduction in transferrin endocytosis in cells expressing G2019S, which indicates disruption of endocytic protein recruitment near the plasma membrane. Overall, this study uncovered multiple dynamic alterations to the LRRK2 protein as a result of the G2019S mutation—all of which could lead to neurodegeneration associated with PD.

## 1. Introduction

Parkinson’s disease (PD) is an age-related disorder that impacts approximately 2% of the population over the age of 65 and is typically characterized by resting tremor, rigidity, and bradykinesia [1,2]. PD patients also present severe neurological pathology, which is believed to occur well before clinical onset of associated symptoms [3]. Over the past decade, numerous genetic markers have been identified and linked to PD [4,5,6,7,8,9,10]. Mutation of one specific gene, *Leucine-Rich Repeat Kinase 2* (*LRRK2*), has been noted to cause both clinical and neuropathological phenotypes [11]. The LRRK2 protein (~280 kDa) contains multiple domains, which are organized with N- and C-terminal protein interaction domains and a central enzymatic core [12,13]. Several reports have confirmed that LRRK2 contains both GTPase and kinase activity with a number of PD causing mutants found to cluster within the central catalytic region [14,15,16,17,18,19,20]. The most prevalent LRRK2 mutation, G2019S, has increased kinase activity and decreased GTPase activity, indicating that LRRK2 enzymatic activity may be linked to PD pathogenesis [21,22,23,24]. Defining a molecular mechanism of LRRK2 activity is essential to understanding its role in PD etiology.

LRRK2 has been reported to exist as both cytosolic and membrane-bound forms localized to cellular structures, including around caveolae, microvilli, multivesicular bodies, and autophagic vesicles [25,26]. The presence of distinct membrane structures containing LRRK2 indicates a potential role in the regulation of membrane protein dynamics [27,28,29,30,31,32,33]. Recently, multiple membrane proteins, such as Rabs and Endophilin A1 (EndoA1), were reported to have direct interactions with or as kinase substrates for LRRK2 [34,35]. Rabs are a large family of GTPase proteins that regulate transitions through phases of the vesicle cycle, such as from clathrin-coated pits to functional uncoated synaptic vesicles, from early to late endosomes, and protein sorting from the ER to the Golgi [27,28,29,31,36,37,38,39,40,41,42,43]. Dysregulation of Rab vesicular pathways have also been reported as a mechanism for neurodegeneration within PD [44]. Based on this distribution and these hetero-interactions, LRRK2 is predicted to be involved in several cellular processes that include vesicle formation and trafficking.

LRRK2 is a member of the family of ROCO proteins—most of which have been proposed to require GTPase-mediated dimerization to activate the kinase domain [13,24,45,46]. There is a large amount of evidence that LRRK2 can self-associate, both in solution and cells, which suggests the possibility of self-association as a mechanism to regulate LRRK2 function [13,28,29,40,46,47,48]. The oligomerization of LRRK2 throughout different subcellular structures implies a spatial dependence of activity where the membrane-bound oligomerization may increase kinase activity. Consistent with this, a previous report suggested that membrane-bound LRRK2 was dimeric and catalytically more active than monomeric LRRK2 within macrophage-like cells [49]. However, questions remain as to whether dimerization is a mechanistic control of LRRK2 protein function as well as if PD pathogenic variants of LRRK2 disrupt these dynamic fluctuations.

To help define the role of LRRK2 self-association and the regulation of subcellular LRRK2 distribution, we utilized advanced fluorescence microscopy methods to examine the spatial dynamics of LRRK2. In order to characterize the alterations in LRRK2 protein homo- and hetero-interactions in response to the kinase activating PD mutation G2019S, we used fluorescence fluctuation spectroscopy (FFS), fluorescence lifetime imaging microscopy (FLIM) with Förster resonance energy transfer (FRET), and total internal reflection fluorescence (TIRF) microscopy. Through these measures, G2019S was found to stabilize homo-interactions within the cytosol, near the plasma membrane, and likely with cytosolic vesicles. This enhanced ability to oligomerize was correlated with a reduced endocytosis profile of cells expressing G2019S. Specifically, the G2019S mutation altered the rhythmic removal of vesicles from the plasma membrane resulting in a reduction in transferrin endocytosis. G2019S-expressing cells were also found to have increased complex formation with EndoA1 and improper clathrin puncta dynamics. These changes to LRRK2 protein dynamics likely contribute to aberrant cell functioning and to the development of PD pathology. Overall, our results provide further evidence that supports a pathway in which alterations to the kinase activity of LRRK2 alters overall protein self-association and ultimately disrupts the regulation of proper endocytosis.

## 2. Results

### 2.1. Cytosolic G2019S Has a Higher Propensity to Form Stable Oligomers and to Associate with Vesicles

**Figure 1:**
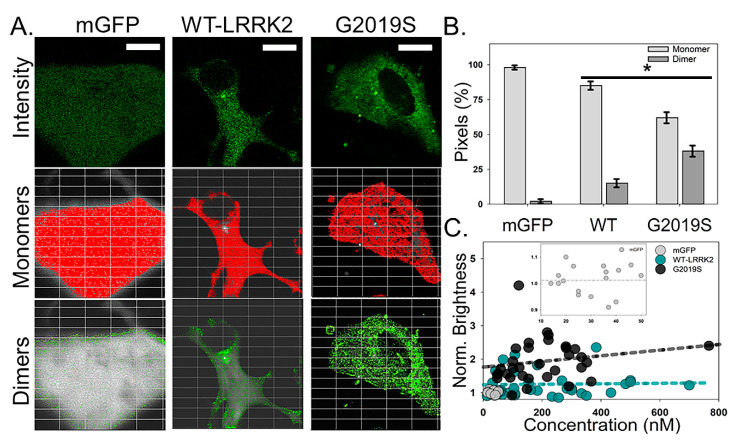
G2019S LRRK2-GFP has increased self-association within the cell body. (**A**) Average intensity images of mGFP, WT LRRK2-GFP, and G2019S LRRK2-GFP collected using two-photon (2P) confocal N&B conditions. Scale bar is 20 µm. Overlay of pixels associated with monomers (red) and dimers (green) onto the corresponding representative intensity images. (**B**) Average pixel percentage of monomers and dimers for mGFP, WT, and G2019S. Statistical analysis was performed using a two-sample *t*-test (*p* < 0.05). Error bars represent the S.E.M. *n* ≥ 20 cells. (**C**). The oligomeric state of WT (dark cyan) and G2019S (black) within the cytosol as a function of concentration. The best-fit line for each construct is highlighted on the graph as a dashed line. The control condition, mGFP (light gray dash), is highlighted within the inset for visual comparison to our monomeric standard.

To distinguish differences between WT and G2019S LRRK2, we transfected differentiated SHSY-5Y cells with WT and G2019S constructs. Figure 1A shows the intensity distribution for cells expressing WT or G2019S. From the autocorrelation function (ACF), we determined that all analyzed samples were expressing fluorescent LRRK2 constructs at a concentration of less than 300 nM (WT = 205 ± 85 nM and G2019S = 175 ± 100 nM; (Figure 1A, Table 1). As expected, WT shows an intensity pattern that is diffused throughout the cytosol with minimal fluorescence intensity within the nucleus. However, G2019S transfected cells contain visible bright puncta as well as notable fluorescence signals from the nucleus (Figure 1A). Number and brightness (N&B) analysis and photon counting histogram (PCH) were used to quantify the oligomerization state in the cell body (Table 1). Both WT and G2019S-expressing cells contain primarily monomeric LRRK2 within the cell body (Figure 1A). However, brightness analysis demonstrated a significant increase in oligomer density of G2019S protein compared to WT (Figure 1B). Normalized brightness values indicate that the predominant freely diffusing species of G2019S protein throughout the cytosol is on average a dimer or larger (Table 1). We note that self-association of WT was not dependent upon concentration (up to ~600 nM, Figure 1C) while G2019S showed a non-significant trend associated with concentration (*R*^2^ = 0.035).

**Figure 2:**
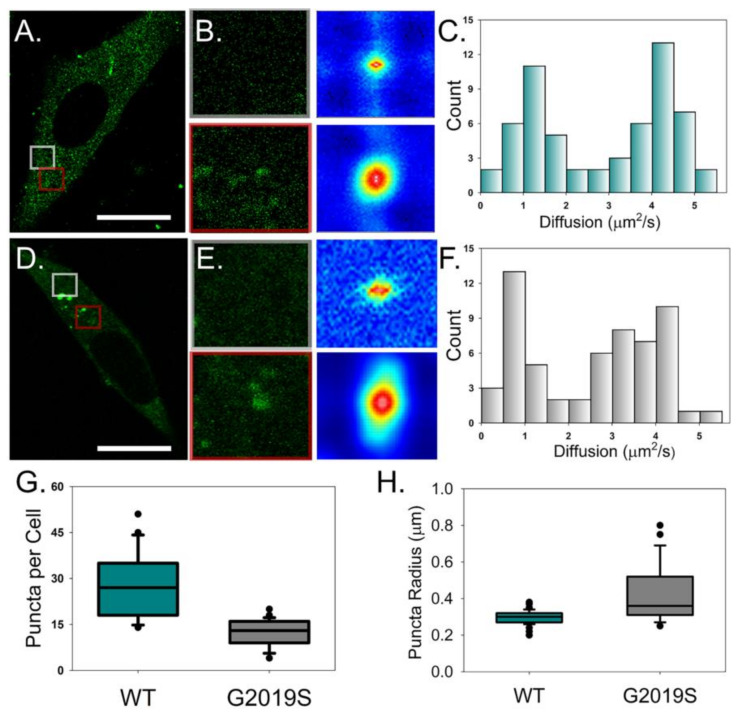
Fluctuation analysis reveals multiple populations of diffusing LRRK2-GFP. (**A**,**D**) A representative average intensity image of WT LRRK2-GFP (**A**) and G2019S-GFP (**D**) collected using 2P confocal microscopy and fluctuation analysis conditions. Scale bar is 15 µm. (**B**,**E**) Intensity images (left column) and autocorrelation functions (ACF) (right column) corresponding to the colored boxed ROIs (grey: top, red: bottom) from the full cell image. (**C**,**F**) Average histogram of diffusion rates obtained for WT LRRK2-GFP (**C**) and G2019S-GFP (**F**). Box plots of WT LRRK2-GFP (dark cyan) and G2019S LRRK2-GFP (gray) comparing the number of puncta per cell (**G**) and cytosolic puncta radius (**H**). Statistical analyses were performed using a two-sample *t*-test (*p* < 0.05). *n* ≥ 20 cells per condition.

Spatial autocorrelation analysis yielded a diffusion rate slower than expected for both WT and G2019S, along with a high standard deviation between cells (Table 1). The transition from monomer to dimer is not expected to cause a significant decrease in diffusion. However, we instead observed bright puncta throughout the cytosol that fluctuated within our region of interest (ROI) and were primarily observed around the nucleus (Figure 2A,B). When examined over time, these bright spots showed rapid movement and directionality. This suggests that these fluorescent LRRK2 particles are most likely bound to vesicles and not caused by freely diffusing protein oligomers. To address the contribution of these particles to our fluctuation signal, we analyzed our fluctuation data using a combination of bimodal fitting and analyzed regions within the cytosol independently. This resulted in two unique populations of diffusing WT: a faster (4.1 µm^2^/s) population with diffusion rates similar to monomer/dimer protein (grey ROI) and a slower rate (1.2 µm^2^/s) indicative of WT bound to membranes (red ROI) (Figure 2B,C). Even though there was no change to the average diffusion of G2019S when compared to WT, this modular diffusion analysis found vastly different profiles of LRRK2 populations between conditions. Unlike WT-expressing cells, the freely moving state of G2019S is indicative of a dimer-tetramer construct (3.5 µm^2^/s), while the slow-moving population had a significant reduction in the rate of diffusion to 0.67 µm/s (*p* < 0.01). Further characterization of these puncta confirmed that while G2019S-expressing cells have significantly fewer spots per cell, these areas are both larger in size and are associated with a much higher brightness value (i.e., more bound LRRK2 protein) than WT (Figure 2D–F). These results support a model of LRRK2 stabilization when G2019S undergoes protein self-association. This stabilized form may promote the targeting of LRRK2 to membrane structures or stabilize LRRK2 when the protein is associated with cellular membranes.

### 2.2. Membrane-Bound G2019S Forms Spatially Distinct Clusters that Show Large Fluctuations in Fluorescence Intensity

Multiple reports have provided evidence that LRRK2 activity is enhanced when in a dimer state [13,17,24,46]. In addition, our previous study of LRRK2 oligomerization in living cells using fluorescence fluctuation spectroscopy (FFS) showed that LRRK2 is predominantly monomeric in the cytosol and predominantly dimeric when bound to the plasma membrane [46]. This model of activity is appealing given that the cellular locations of many identified LRRK2 substrates are within distinct compartments. However, it is unclear what role altered kinase activity, such as with the G2019S mutation, has on recruitment or stabilization of LRRK2 self-association in specific subcellular structures. To address this question, we utilized TIRF microscopy to measure the dynamics of WT and G2019S near the plasma membrane.

**Figure 3:**
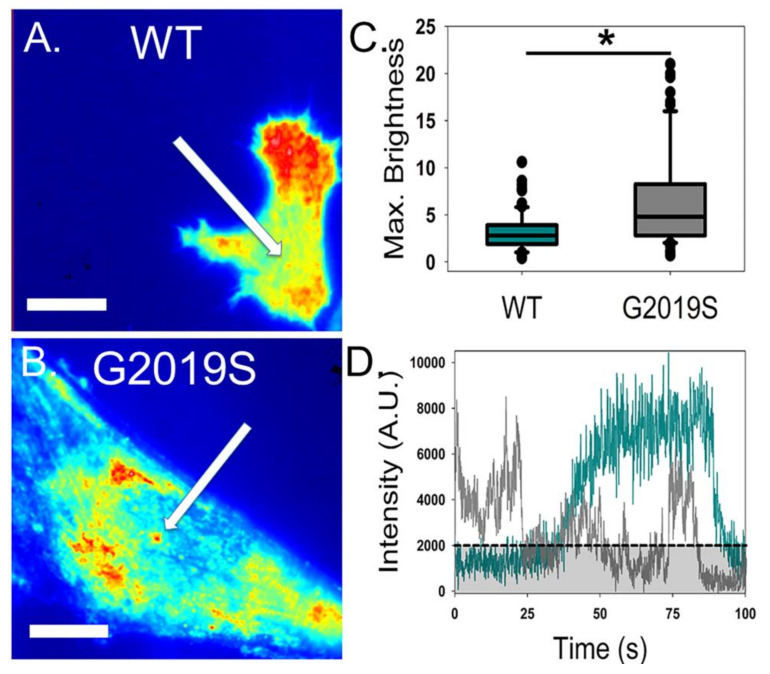
G2019S LRRK2-GFP has altered distribution and time dependence near the plasma membrane. Average total internal reflection fluorescence (TIRF) microscopy intensity images for WT LRRK2-GFP (**A**) and G2019S LRRK2-GFP (**B**), with arrows depicting clustering of fluorescent molecules. Color within this figure represents the level of intensity detected by the CCD, which is scaled proportionally from lowest (blue) to the maximum intensity (red). (**C**) Comparison of the largest detected fluorescent component near the plasma membrane of WT and G2019S-expressing cells (*p* < 0.05). (**D**) Representative fluctuation of fluorescence intensity at a puncta during a 100 s time period for WT (dark cyan) and G2019S (gray). The black line represents minimal intensity change for inclusion of a fluctuation event. Scale bars are 12 µm. *n* ≥ 40 cells per condition.

Cells expressing either WT or G2019S produced strong intensity images, demonstrating that there is likely an association of these fluorescent constructs with the plasma membrane (Figure 3A,B), which is consistent with our previous findings [46]. Fluctuation analysis indicated that under our transfection conditions, the plasma membrane-associated concentration was similar for both variants (Table 2). While the average brightness did not differ between groups near the plasma membrane, membrane-associated G2019S had a slight but significantly higher percentage of pixels associated with dimeric LRRK2 when compared to WT (Figure S1). However, there was no significant difference in the number of pixels associated with monomeric state or with an oligomerization state higher than a dimer (Figure S1).

We also noted that G2019S-expressing cells showed more specific localization of fluorescence intensity than the more homogenous pattern of WT, indicating the presence of abnormally bright fluorescent puncta (Figure 3A,B). The presence of these bright puncta biases the brightness calculation due to their large contribution to the variance, resulting in a skewed quantification of G2019S clustering. Therefore, we compared the brightest species detected near the plasma membrane as an attempt to compare membrane-associated differences between constructs. Though this measurement is not accurate in determining the actual oligomeric state of the protein, it provides a method to analyze the localization differences between constructs. Our analysis confirmed that membrane-associated G2019S contained bright puncta that were significantly (*p* < 0.05) larger than anything observed in WT-expressing cells (Figure 3C). These dense G2019S puncta also showed altered temporal behavior of protein near the plasma membrane when compared to WT (Figure 3D). Specifically, G2019S puncta showed large variances and drastic changes in intensity over the 100 second examination period, whereas the WT protein underwent smooth build-up and decline near the plasma membrane (Figure 3D). Overall, the promotion of protein clustering and alteration to the spatial dynamics of LRRK2 associated with the plasma membrane due to G2019S expression noted in these experiments indicates a potential disruption of membrane mechanisms. To determine the net function of LRRK2 on plasma membrane vesicle formation, we used ccFFS and FRET to quantify potential disruption in endocytosis due to the G2019S mutation.

### 2.3. The G2019S Mutation Disrupts Endocytosis by Changing Endocytic Protein Membrane Dynamics

**Figure 4:**
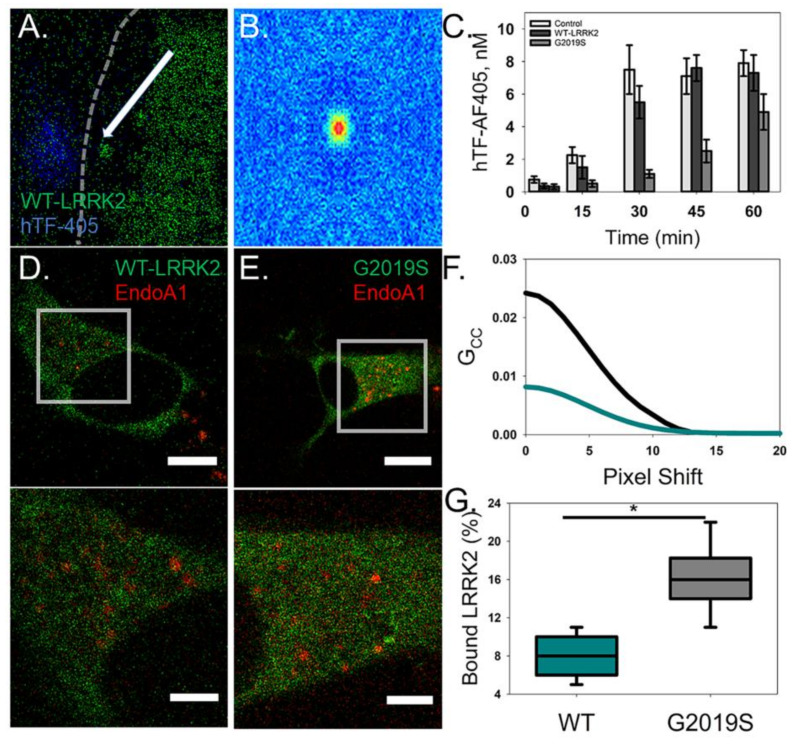
G2019S-expressing cells show a reduction in endocytosis of human transferrin. (**A**) A representative image of a WT LRRK2-GFP (green)-expressing cell treated with hTF-AF405 (blue). Arrow depicts a LRRK2 bound vesicle that moved to and from the site of the membrane (dashed white line) with clustered hTF-AF405. (**B**) Cross-correlation raster image correlation spectroscopy (ccRICS) autocorrelation function (ACF) of LRRK2 and hTF. (**C**) Control and transfected cells were treated with hTF-AF405 and the amount of internalized hTF-AF405 was determined, using RICS, at the designated time points. Error is shown as the S.E.M. (**D**,**E**) Representative intensity images of cells co-expressing EndoA1-mCherry (red) with either WT LRRK2-GFP (left; green) or G2019S LRRK2-GFP (right; green). Scale bar is 15 µm. ROI selected for ccRICS analysis corresponding to the boxed regions within the full cell intensity images. Scale bar is 3 µm. (**F**) Representative fits of the ccRICS ACF for WT (dark cyan) and G2019S (black) with EndoA1-mCherry. (**G**) The amount of LRRK2 interacting with EndoA1-mCherry as a percentage of total protein (*p* < 0.05).

In order to evaluate the effect of G2019S mutation on endocytosis, transfected cells were imaged with fluorescently labeled transferrin (hTF-AF405). Confocal imaging captured the LRRK2 constructs being targeted to and leaving membrane regions containing clusters of hTF-AF405 (Figure 4A). The spatial ACFs for these clusters containing LRRK2 were not symmetrical, indicating that their movement is directional and not solely due to passive diffusion (Figure 4B). Furthermore, time-course experiments comparing the cytosolic concentration of hTF-AF405 showed that G2019S-expressing cells have delayed hTF-AF405 endocytosis (Figure 4C). These differences could be due to a change in endocytosis function due to G2019S mutation. To explain why endocytosis is being affected, we co-transfected cells with EndoA1, a known LRRK2 substrate that regulates vesicle endocytosis [43]. Fluctuation analysis confirmed that WT is able to form a complex with EndoA1 although the amount of protein within the complex was only a small percentage of the total amount of LRRK2 in the cell (Figure 4D,F,G). Interestingly, G2019S transfected cells produced larger interaction signals indicating an increased percentage of G2019S complexes with EndoA1 when compared to WT (Figure 4E–G). This finding was further supported by FRET analysis, which demonstrated a direct interaction between LRRK2 and EndoA1 constructs due to the distance constraint associated with producing a FRET signal (Figure S2). This finding points to potential functional alterations in the endocytosis mechanism due to the G2019S mutation.

**Figure 5:**
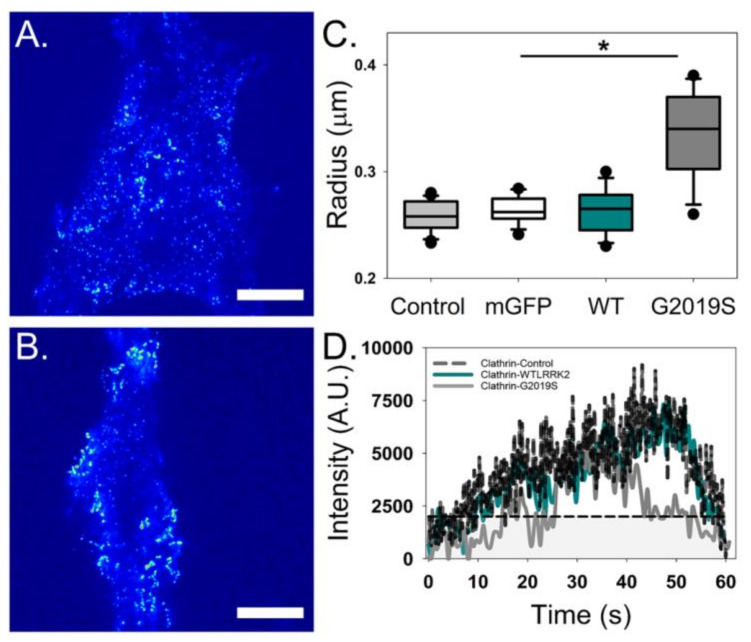
Clathrin puncta on the plasma membrane are altered when co-transfected with G2019S. Total internal reflection fluorescence (TIRF) microscopy intensity images of clathrin puncta co-expressed with either WT LRRK2-GFP (**A**) or G2019S LRRK2-GFP (**B**). (**C**) Clathrin expressed with G2019S shows a significant increase in the radius of endocytic spots when compared to controls and WT (*p* < 0.05, *n* ≥ 40 cells). (**D**) A representative intensity fluctuation of a single clathrin puncta plotted as a function of time over a 60 s period for the clathrin control (black), WT LRRK2-GFP (dark cyan) and G2019S LRRK2-GFP (grey).

TIRF microscopy of LRRK2-transfected cells co-expressing clathrin-mCherry provided further support to LRRK2 activity in endocytosis. WT-expressing cells were found to have no observable difference in clathrin puncta, including size and dynamics, as compared to those in control cells expressing clathrin without heterologous LRRK2 (Figure 5). In contrast, clathrin puncta were significantly larger and showed irregular intensity fluctuations when co-transfected with G2019S (Figure 5C,D). These data indicate that G2019S expression disrupts proper regulation of clathrin formed vesicles during endocytosis, potentially by reducing recruitment of adaptor proteins required for vesicle fission such as Endophilin. Taken together, these results demonstrate a definitive interaction between LRRK2 and EndoA1 as well convincing evidence supporting the role of G2019S in the disruption of endocytosis. These differences could potentially explain the presence of abnormally shaped vesicles in the presynaptic terminal that are characteristic of PD [42,47,50].

## 3. Discussion

The molecular mechanisms that govern the enzymatic properties of LRRK2 are critical to understanding LRRK2 mediated neurodegeneration. PD has been associated with abnormalities in endocytosis and vesicle formation which result in a build-up of dopamine in the presynaptic terminal and a deficit of available dopamine in the synapse [51,52,53]. It has been proposed that LRRK2 is a regulator of both endocytosis and vesicle trafficking due to multiple direct interactions with substrate proteins linked to these processes [27,28,29,31,33,34,37,40]. These interactions between LRRK2 and endocytic proteins suggest a potential role for LRRK2 in regulating endocytosis at the plasma membrane. The inherent changes to the dynamics and self-association of LRRK2 due to PD-associated mutations are critical to understand in order to advance targeted therapeutics. We have quantitatively measured the distribution and dynamics of WT, and the PD mutant variant G2019S within live cells using fluctuation and FRET microscopy. Our utilization of multiple quantitative, live-cell microscopy methods has allowed us to examine the unique properties of LRRK2 and provided mechanistic insight of how increased kinase activity alters cellular properties that could lead to neurodegeneration.

### 3.1. Alterations in Enzymatic Activity Might Be Linked to Stabilizing LRRK2 Oligomers

LRRK2 is known to exist within multiple subcellular domains, and the protein oligomerization is different between these structures [46,49]. The G2019S mutant, which has higher kinase activity compared to WT, was observed to form a larger amount of homo-interactions throughout the cell, including puncta structures near the plasma membrane. Moreover, we were able to attribute these differences to an enhanced propensity for the G2019S mutation to self-associate in a manner that is not dependent on protein concentration. These findings stress that the enzymatic activity could have an important partnership with LRRK2 oligomerization. Alterations to catalytic domains (either GTPase or kinase) could cause spatial disruptions for LRRK2 homo-interactions in which assembly and disassembly are deregulated and could potentially allow for PD variants, such as G2019S, to bypass normal enzymatic regulation. Even though this pathway needs further examination, it is worth considering that perhaps the increased kinase activity of the G2019S mutant stems from a larger pool of available active dimer species when compared to WT-LRRK2. Overall, our results show that the G2019S mutation has a higher tendency to self-associate, which provides new insight into the mechanism behind the notably increased kinase output of this mutation.

### 3.2. G2019S Mutation Disrupts LRRK2 Recruitment to Intracellular Membranes

Previous work has indicated that LRRK2 phosphorylates multiple members of the Rab GTPase family and that Rabs may also function to recruit LRRK2 protein to cellular structures [53]. This concept is highlighted by the findings that pathogenic mutations of LRRK2 correlate with disperse Golgi membranes [54]. Our findings tie in well with this model as we were able to quantify, through trajectory and diffusion, both free and vesicle-bound populations of LRRK2. Cells transfected with G2019S were characterized as having fewer and larger intracellular vesicles, which contained more LRRK2 protein compared to WT-expressing cells. Changes in LRRK2 dynamics may result in functional differences to the vesicle cycle, as noted by changes in LRRK2 substrate interactions or a preference of dimerized LRRK2 to localize to membrane structures. This deviation would be expected to promote the recruitment of oligomerized LRRK2 to targeted structures (such as vesicles), which would extend the retention time and interaction with kinase substrates. It is likely that the increased kinase output of G2019S is a causative factor of the altered substrate protein interaction. Rather than simply causing increased levels of self-association, the G2019S mutation could instead reduce the ability of LRRK2 to dissociate from these endosomal membranes.

### 3.3. G2019S Expression Causes Alterations in Endocytic Protein Dynamics

PD has been associated with abnormalities in endocytosis and vesicle formation, which is consistent with findings that suggest an interaction of LRRK2 with membrane structures [27,29,38,41,42,55]. Our data emphasize these previous observations, as we were able to monitor the movement of LRRK2 to sites of endocytosis near the plasma membrane. It is possible that these data are indicative of cargo-containing vesicles which retain LRRK2 protein during entry back to intracellular compartments. Furthermore, we were able to determine that the G2019S mutation disrupts the endocytic process by hindering and changing membrane dynamics through increasing LRRK2 protein clustering near the membrane. This observation was further supported by the notable abnormally enhanced interaction with EndoA1. Such alterations in clathrin-mediated endocytosis were characterized by disruption in hTF internalization and clathrin puncta dynamics on the plasma membrane. These findings are consistent with what is known about the alterations of synaptic vesicles during PD pathology [28,33,47]. It has been established that G2019S does in fact have a lower GTPase activity when compared to WT-LRRK2, which slows the transition from active state dimers to dissociation into monomer species [15,24,56]. It is possible that this increased interaction disrupts the normal membrane association of EndoA1 leading to reduced recruitment to clathrin necks and/or the abnormally shaped vesicles that are associated with PD [42,47,50]. Overall, our data suggest that the function of altered G2019S oligomerization is most likely due to an increased propensity to self-associate, which may be exacerbated by an inability to dissociate from cellular membranes due to lowered GTPase activity. While this pathway requires more clarification, it is important to note that these changes are specifically related to PD neurodegeneration because EndoA1 is primarily expressed in neuronal tissues [32,55]. Therefore, EndoA1 poses a unique therapeutic avenue for LRRK2-mediated PD in order to target only neuronal cells and limit off target side effects.

## 4. Conclusions

Ultimately, understanding the dynamic alterations to LRRK2 under cellular conditions is an essential first step in designing advanced targeted PD therapeutics. While there has been a lot of promising work in the development of LRRK2 kinase inhibitors for a novel disease-modifying therapy, determining the mechanistic changes to LRRK2 due to PD-associated mutation will help to define the cellular processes affected by these treatments. The results discussed throughout this article were collected to narrow this gap in knowledge. In conclusion, it is likely that the promotion of protein stabilization and increased self-association are key mediators in regulating the enzymatic alterations and spatial organization of pathogenic LRRK2. These properties are likely the cause of increased phosphorylation of kinase substrates and the aberrant membrane dynamics that are characteristic of LRRK2 mediated PD. Overall, we believe that the novel dynamic findings discussed in this study will provide valuable insight into the mechanistic properties of G2019S LRRK2 and progress the field toward novel targeted disease-modifying therapies for PD.

## 5. Materials and Methods

### 5.1. Cell Culture and Plasmid Transfection

SH-SY5Y cells (Cat. 94030304, ATCC, Manassas, VA, USA) were maintained at 37 °C and 5% CO_2_ in tissue culture-treated T75 flasks (Cat. 315, Corning, Corning, NY, USA) using DMEM/F12 (phenol red free) with 15 mM HEPES and L-glutamine (Life Technologies, Grand Island, NY, USA) and added 1× Antibiotic-Antimycotic, 1 mM sodium pyruvate, 1× MEM NEAA (all from Gibco, Thermo Fisher Scientific, Waltham, MA, USA), and 20% FBS (Atlanta Biologicals, Flowery Branch, GA, USA). Cells were split at 80% confluency using 0.08% trypsin-EDTA in PBS (Gibco, Thermo Fisher Scientific, Waltham, MA, USA). Prior to imaging, cells were plated onto glass-bottom dishes (14 mm uncoated for two-photon (2P) confocal microscopy and 10mm collagen coated for TIRF microscopy, MatTek, Ashland, MA, USA) and were differentiated into dopaminergic neuron-like cells using MEM with Earle’s salts (Life Technologies, Grand Island, NY, USA) supplemented with 10 mM HEPES, 1× Antibiotic-Antimycotic, 1 mM sodium pyruvate, 1× MEM NEAA (all from Gibco, Thermo Fisher Scientific, Waltham, MA, USA), 5% FBS (Atlanta Biologicals, Flowery Branch, GA, USA), and 1 mM retinoic acid (RA) (Sigma Aldrich, St. Louis, MO, USA) using a 10 day differentiation protocol established by Korecka and colleagues [57]. Cell culture media was changed to RA differentiation media when dishes were 20% confluent (day 1) and media changes were performed on days 1, 3, and 6. LRRK2 transfection occurred on day 8 using the LRRK2-GFP BacMam vectors for WT and G2019S at 2% concentration for confocal imaging and 3% concentration for TIRF experiments by following the manufacturer’s suggested protocol (Invitrogen, Carlsbad, CA, USA). For experiments using co-transfection and for transfection of control plasmids, Lipofectamine™ 2000 was used on day 9 with 0.5–3 μg of DNA plasmid per dish following the manufacturer’s suggested protocol (Invitrogen). Imaging experiments were performed on day 10 for data collection. To avoid overexpression, mGFP was transfected for only 4 h on the day of imaging.

### 5.2. Labeling Transferrin for Endocytosis Experiments

Human serum transferrin (hTF; Biogems, Westlake Village, CA, USA) was labeled with Alexafluor 405 NHS ester (hTF-AF405) following the manufacturer’s suggested protocol. Briefly, 10 mg of hTF was dissolved in 20 mM sodium bicarbonate, pH 8.3 and incubated with Alexafluor 405 (1:10 protein/label ratio) for 1 h at room temperature. The reaction was quenched with 1.5 M hydroxylamine, pH 8.5. The reaction mixture was dialyzed against 20 mM HEPES, pH 7.5 containing 150 mM NaCl for 24 h prior to use.

### 5.3. Fluctuation and FLIM/FRET

Fluctuation data were collected using an Alba fluorescence correlation spectrometer (ISS, Champaign, IL, USA), connected to a Nikon TE2000-U inverted microscope (Nikon, Melville, NY, USA) with *x*-*y* scanning mirrors and a PlanApo VC 60 × 1.2 NA water objective as previously described [58]. The 2P excitation was provided by a Chameleon Ultra (Coherent, Santa Clara, CA, USA) tuned to 920 nm for both single GFP emission and FRET collection, while 1000 nm was used for co-transfection experiments to obtain optimal excitation of both fluorophores. A 680 nm short-pass filter (FF01-680; Semrock, Rochester, NY, USA) and dichroic mirror (700dcxru, Chroma, Bellows Falls, VT, USA) was used to spectrally filter the emission of the fluorophores with separate photomultiplier tubes. Cells were imaged using a temperature and humidity-controlled stage at 37 °C with an objective wrap heater (Tokai Hit, Fujinomiya, Shizuoka, Japan) to mimic the environment of the incubator and minimize temperature drift.

Photon counting histogram (PCH) analysis was used to obtain dynamic information at a single arbitrary location, which was chosen in a region of the cytosol that was not near the plasma membrane or the nucleus. Fluctuations in fluorescence intensity were monitored for a period of 4 min at a 50,000 Hz sampling rate for each cell. Average brightness values were analyzed with the Vinci software while integrating the detector dead-time of 50 ns [59,60].Variations in molecular brightness that occur day to day due to changes in equipment properties was controlled for by utilizing a monomeric EGFP standard (both in cells and in solution) at numerous concentrations to obtain an average monomer value for each trial. Experimental data were then compared to this standard value for determining proper oligomerization values.

Raster image correlation spectroscopy (RICS) was also used to examine the potential alterations in protein dynamics [61,62]. In short, we selected 12.8 μm (50 nm/pixel) regions of interest (ROIs) for each cell that encompassed the majority of the cytosol while attempting to avoid the cell nucleus and plasma membrane. Each cell analyzed consisted of a frame size of 256 × 256 pixels, a pixel sampling time of 12.5 μs, and measurement of 100 frames (approximately 1 min of sampling time per cell). Beam waist (ω_0_) calibration was performed daily before each experiment by utilizing the GFP solution standard and was consistently recorded at approximately 0.35–0.4 µm.

Fluorescence lifetime imaging microscopy (FLIM) measurements were obtained with an ISS A320 FastFLIM box with photomultiplier detector joined to the Ti:Sapphire laser that created 80 fs pulses at a repetition rate of 80 MHz (H7422P-40, Hamamatsu, Hamamatsu City, Japan) as previously described [63]. The fluorescence signal was filtered from the excitation light using a 520 nm bandpass filter (FF01-520/35; Semrock Rochester, NY, USA) that was secured in front of the detector. Purified GFP in a filtered 5% BSA/PBS solution was used for standardizing lifetimes at 2.95 ns. Using this method, the fluorescence lifetimes associated with each pixel are plotted as previously described. If energy transfer occurs, the pixels will move into the universal circle due to a shortening of the donor lifetime. During co-transfection experiments, a control plasmid (mCherry) was co-transfected with the LRRK2 constructs to establish that the interaction was not due to the fluorescent protein interacting with our proteins of interest (data not shown).

### 5.4. Total Internal Reflection Fluorescence (TIRF) Imaging

Data for protein dynamics associated with the plasma membrane were collected using a Nikon Eclipse Ti TIRF microscope with a 60 × 1.45 NA oil objective which we previously reported [58]. One-thousand frames were collected per cell with a cascade 512B EMCCD camera (Photometrics, Tucson, AZ, USA). Each protein was excited independently (EGFP at 488nm and mCherry/mRFP at 543 nm) via a triple band excitation filter (405/488/594 nm; Chroma, Bellows Falls, VT, USA) within the infinity space.

### 5.5. Confocal Imaging of hTF

Fluctuation imaging of hTF endocytosis was performed on an Olympus Fluoview FV1000 as previously described [64]. Briefly, fluctuation data for hTF-AF405 and LRRK2-GFP were collected using excitation at 405 and 488 nm with 100 intensity images being obtained at 20 μs/pixel. Cells were incubated on the microscope where hTF-AF405 was added to the cells and imaged at 0, 15, 30, 45, and 60 min.

### 5.6. Data Analysis

VistaVision software (ISS, Champaign, IL, USA) was used to analyze PCH data. SimFCS (obtained from the Laboratory for Fluorescence Dynamics) was used to analyze data from RICS, N&B, and FLIM/FRET experiments. Descriptive details on analysis via these software packages have been previously described [46,62,65]. For Number and brightness (N&B), the percentage of pixels was calculated individually for each cell as the number of pixels associated with the brightness value of interest divided by the total number of pixels with a positive GFP signal.

### 5.7. Statistical Analysis

All statistical analysis was performed using SAS University (Cary, NC, USA). Data were analyzed using a two-sample *t*-test at an alpha level of 0.05 for each experiment. Each N is defined as the average experimental value of all cells collected over a single experiment.

## Figures and Tables

**Table 1:** Confocal fluctuation analysis of cytosolic LRRK2-GFP constructs.

Protein ^a^	Concentration (nM)	Average Diffusion (µm^2^/s)	Average ε (cpsm)	Normalized ε ^c^
mGFP	30 ± 10	9.0 ± 2.0	35,000 ± 1,000	1.0 ± 0.1
mLRRK2; dLRRK2; tLRRK2	N.A.	4.9	35,000	1.0
		4.0	70,000	2.0
		2.9	140,000	4.0
WT LRRK2-GFP	205 ± 85	2.4 ± 1.1	43,000 ± 9,000	1.2 ± 0.3
G2019S LRRK2-GFP	175 ± 100	2.0 ± 0.9	72,000 ± 15,000 ^b^	2.1 ± 0.5

^a^—For each condition (mGFP, WT LRRK2-GFP, G2019S LRRK2-GFP) with calculated concentrations of transfected proteins (*n* ≥ 20 cells), no significant trend was found for concentration and thus each concentration was included in the final calculations. Monomeric (mLRRK2), dimeric (dLRRK2), and tetrameric (tLRRK2) are calculated theoretical values for the purpose of comparing cytosolic properties of the experimental conditions. Therefore, there are no reportable concentrations for these species. ^b^—*p* < 0.05 from a two-sample *t*-test when control sample, mGFP, was compared to the experimental conditions. ^c^—Normalized brightness values were generated by dividing the brightness of the sample over the monomer control. Error is reported as ± standard deviation.

**Table 2:** TIRF fluctuation analysis of plasma membrane-associated LRRK2-GFP.

Transfected Protein(s)	Concentration (nM)	Average Brightness (cpsm)	Norm. Brightness	Puncta Variance (sec)
mGFP	100 ± 10	1.10 ± 0.03	1.0	N/A
WT LRRK2-GFP	55 ± 15	1.33 ± 0.08	2.5	55 ± 15
G2019S LRRK2-GFP	90 ± 30	1.33 ± 0.10	2.5	25 ± 10

Average brightness (ε) is reported as counts per second per molecule. Normalized brightness values were generated by dividing the brightness of the sample over the monomer control. Puncta variance refers to the average time spent on the plasma membrane. Error is reported as ± standard deviation. *n* > 20 cells per condition.

## Supplementary Materials

Supplementary Materials are available online.
