# Supplementary material for: Fluctuation Imaging of LRRK2 Reveals that the G2019S Mutation Alters Spatial and Membrane Dynamics
Source: Molecules. 2020 May 31;25(11):2561. doi: 10.3390/molecules25112561 (PMC7321188; doi:10.3390/molecules25112561)
Supplement: Supplementary file 1 [file molecules-25-02561-s001.pdf]

Supplementary Materials

Fluctuation Imaging of LRRK2 Reveals That the G2019S Mutation Alters Spatial and Membrane Dynamics

Bethany J. Sanstrum, Brandee M. S. S. Goo, Diana Z. Y. Holden, Donovan D. Delgado, Thien P. N. Nguyen, Kiana D. Lee and Nicholas G. James *

Department of Cell and Molecular Biology, University of Hawaii at Manoa, 651 Ilalo Street, Honolulu, HI 96813, USA; BSANSTRUM@augusta.edu (B.S.); BGOO@augusta.edu (B.G.); dianazyh@berkeley.edu (D.H.); dddelgad@hawaii.edu (D.H.); thienn@hawaii.edu (T.N.); kiana.lee2@wsu.edu (K.L.);

* Correspondence: njames4@hawaii.edu; Ph: 808-956-8332; Fax: 808-692-1970

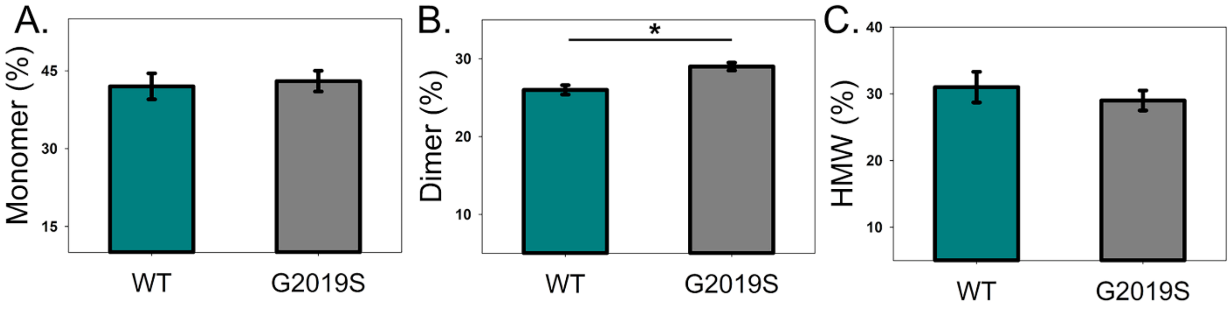

Figure S1. G2019S LRRK2-GFP is associated with significantly higher levels of dimer on the plasma membrane. Bar graphs of average self-association percentages of monomer (**a**), dimer (**b**), and higher molecular weight (HMW) species (**c**) as a function of contribution to total number of pixels per image associated with oligomerization values up to 6. Statistical analysis was performed using a two-sample *t*-test at an alpha level of 0.05. n ≥ 50 cells per group.

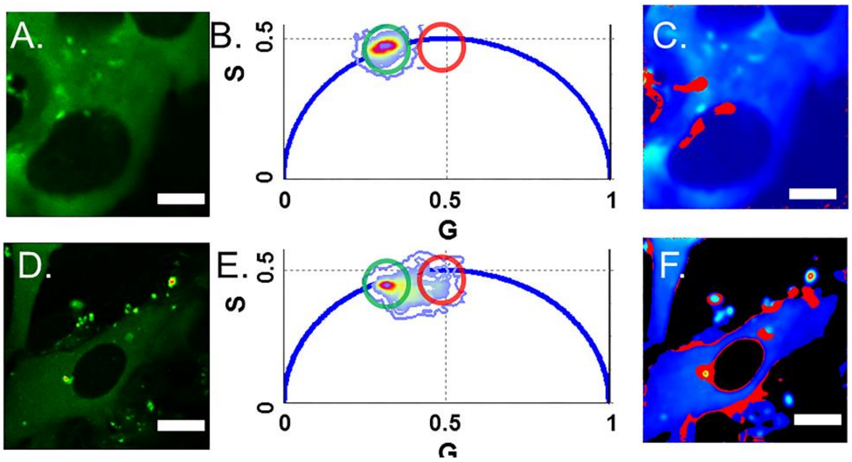

Figure S2. Forster resonance energy transfer (FRET) analysis shows that G2019S LRRK2-GFP has an increased amount of interaction with EndoA1-mCherry. a and d) Fluorescence lifetime imaging microscopy (FLIM) images of WT LRRK2-GFP (top) and G2019S LRRK2-GFP (bottom) when co-expressed with EndoA1-mCherry. Scale bar is 15 μm. b and e) Phasor plots from WT LRRK2-GFP and G2019S LRRK2-GFP containing points associated with GFP lifetime (green circles) and quenched pixels of LRRK2 interacting with EndoA1 (red circles). c and f) Spatial organization of the selected phasor points from (b and e) overlaid onto their corresponding images (a and d).
